# Overlapping Pattern of the Four Individual Components of Dyslipidemia in Adults: Analysis of Nationally Representative Data

**DOI:** 10.3390/jcm13123624

**Published:** 2024-06-20

**Authors:** Wenxiao Zheng, Jiayue Zhang, Ying Jiang, Shuting Wang, Zuyao Yang

**Affiliations:** 1 JC School of Public Health and Primary Care, The Chinese University of Hong Kong, Hong Kong SAR, China; wenxiaozheng@cuhk.edu.hk (W.Z.); wangst@link.cuhk.edu.hk (S.W.); 2 Faculty of Medicine, Macau University of Science of Technology, Macau SAR, China; 3230004421@student.must.edu.mo; 3 Chronic Disease Research Institute, The Children’s Hospital, and National Clinical Research Center for Child Health, School of Public Health, School of Medicine, Zhejiang University, Hangzhou 310052, China; jiangying01@zju.edu.cn

**Keywords:** dyslipidemias, cardiovascular diseases, cross-sectional study

## Abstract

**Background/Objectives**: Dyslipidemia is a well-established risk factor for cardiovascular disease (CVD). However, among available drug treatments, only those targeted at lowering LDL-C and consequently TC have demonstrated efficacy in preventing CVD. This is to say that the benefit for those with isolated high TG or low HDL-C is limited. The objective of this study is to examine the overlapping pattern of the four dyslipidemia components in US adult populations, which is important for quantifying the proportion of those who are less likely to benefit from lipid-lowering drugs and for a more precise use of the drug. **Methods**: A total of 7822 participants aged over 20 with abnormalities in any of the four lipid parameters, excluding those on lipid-lowering medications, were included from the National Health and Nutrition Examination Survey (NHANES) cycles spanning 1999–2000 through 2017–2018. The proportions of different combinations of them were calculated and presented using area-proportional Euler plots. **Results**: High TC, high LDL-C, high TG, and low HDL-C were seen in 32.8% (95% CI: 31.3%–34.2%), 28.1% (95% CI: 26.6%–29.6%), 26.7% (95% CI: 25.4%–28.0%), and 65.9% (95% CI: 64.0%–67.7%) of the people with dyslipidemia, respectively. The proportions of dyslipidemia cases attributable to “high LDL-C or high TC” (irrespective of HDL-C and TG levels), “normal LDL-C, normal TC, but high TG” (irrespective of HDL-C level), and “normal LDL-C, normal TC, normal TG, but low HDL-C” (i.e., isolated low HDL-C) accounted for 37.5% (95% CI: 35.9%–39.1%), 18.3% (95% CI: 17.2%–19.4%), and 44.2% (95% CI: 42.5%–46.0%), respectively. **Conclusions**: Some two-thirds of those with dyslipidemia had low HDL-C or high TG but normal LDL-C and normal TC. As these people are less likely to benefit from currently available drug treatments in terms of CVD prevention, it is important to identify other effective strategies or interventions targeted at them in order to achieve more precise and cost-effective management of dyslipidemia.

## 1. Introduction

Cardiovascular diseases (CVDs) stand as the leading cause of mortality and the main contributor to disability in the United States and globally [1,2,3]. For example, in 2020, CVDs were responsible for 928,741 deaths in the United States, representing approximately one-third of the total number of deaths [2]. Dyslipidemia, defined as the presence of “abnormality” in at least one of total cholesterol (TC), low-density lipoprotein cholesterol (LDL-C), high-density lipoprotein cholesterol (HDL-C), and triglycerides (TG), is a recognized risk factor for CVDs. Specifically, elevated levels of TC, LDL-C, and TG and lowered levels of HDL-C are linked to increased risk for CVDs [4,5,6,7]. The increase in cardiovascular risk associated with a 10 mg/dL increase in TC, LDL-C, and TG ranges from 1% to 5% [8,9], 3% to 14% [10,11,12], and 1% to 7% [13,14,15], respectively. Each 10 mg/dL decrease in HDL-C levels is associated with a 18% to 34% increase in cardiovascular risk [10,12,16].

Among the currently available drug treatments for dyslipidemia, only those targeting LDL-C (which can also lower TC because the main component of TC is LDL-C) have shown consistent efficacy in reducing CVDs and mortality [17,18]. The effectiveness of TG-lowering drugs in terms of CVD prevention is inconclusive, and drug treatments aimed at increasing HDL-C levels do not improve cardiovascular outcomes [19,20,21,22]. Except in the case of very high TG levels that should be addressed promptly to prevent pancreatitis, the 2018 AHA/ACC guidelines make no particular recommendation regarding the treatment for isolated low HDL-C or isolated high TG, but acknowledge these two as important risk-enhancing factors that may favor statin therapy [23]. Although some individuals with isolated low HDL-C or isolated high TG (and normal LDL-C level) may benefit from treatment to further reduce their LDL-C levels, the 2019 ESC/EAS guidelines (page 126) suggest that “Class I recommendation, Level A evidence” for such treatment is only available for those with a 10-year CVD risk > 10% [24], which represents a small fraction of all these individuals [25,26]. In other words, the majority of individuals with isolated low HDL-C or isolated high TG are less likely to benefit from lipid-lowering drug treatment. Therefore, understanding the overlapping pattern of the four individual components of dyslipidemia in adults is important for identifying those who may potentially benefit from lipid-lowering medications, as well as those for whom LDL-C-lowering treatments are less or even not effective. This knowledge can help refine the management strategy and improve the cost-effectiveness of drug treatment for dyslipidemia.

Previous studies have investigated the prevalence of dyslipidemia in the general population of the United States, its variations across different subgroups, and its temporal trends [27,28,29]. However, the overlapping pattern of the four components of dyslipidemia is largely unclear. We thus conducted this study to address this important question by analyzing 20-year data from the National Health and Nutrition Examination Survey (NHANES), a comprehensive cross-sectional survey representing the general adult population of the United States.

## 2. Materials and Methods

### 2.1. Study Population

The data used in this study were obtained from NHANES, which is a series of cross-sectional surveys conducted by the National Center for Health Statistics every two years since 1999. It aims to evaluate the health and nutritional status of the civilian noninstitutionalized population in the United States [30]. The NHANES protocols were approved by the National Center for Health Statistics Research Ethics Review Board. All participants provided informed consent for the utilization of their data. The datasets are publicly available for downloading at https://wwwn.cdc.gov/nchs/nhanes/Default.aspx, accessed on 1 July 2023. The datasets whose names begin with “DEMO”, “LAB13”, “L13”, LAB13AM”, “L13AM”, “TCHOL”, “HDL”, “TRIGLY”, “RXQ_RX”, “RXQ_DRUG”, and “BPQ” were used in this study. The participants who (1) were aged 20 and above, (2) completed a morning fasting blood test during the 10 NHANES cycles from 1999–2000 to 2017–2018, (3) had data on all the four lipid parameters, (4) were abnormal on at least one of the four lipid parameters (see below for definition), and (5) were not taking lipid-lowering drugs, were eligible for this study (*N* = 7822).

### 2.2. Measurements and Definitions

Demographic information, including sex, age, and race, was collected through self-reporting of participants during the interview. Race was categorized as non-Hispanic White, non-Hispanic Black, Mexican American, other Hispanic, and Other/multiracial. The “other Hispanic” group consists of participants who self-reported as Hispanic but not Mexican American. The utilization of lipid-lowering drugs was determined based on participants’ responses to the question BPQ100D in the Blood Pressure/Cholesterol section and Prescription Medications section in the survey. Participants who reported having their blood cholesterol checked (question BPQ060) and having been advised to take a prescription for cholesterol (question BPQ090D) were subsequently asked question BPQ100D, “(Are you/Is SP) now following this advice to take prescribed medicine?”. This question served as a reference for their medication-taking status. In the Prescription Medications section, participants were asked to report all prescription medications they had been taking within the past 30 days. Only participants who answered “no” to BPQ100D and did not mention any lipid-lowering medication in the Prescription Medication section were considered as non-medication users and thus eligible for this study.

Serum lipid profile was measured at mobile examination centers. TC and TG were directly measured by enzymatic reactions. HDL-C was directly measured by the heparin–manganese precipitation method. LDL-C was determined through calculation using the measured values of TC, HDL-C, and TG according to the Friedewald formula [31]. The calculation was valid only when TG < 400 mg/dL. When TG was ≥400 mg/dL, the LDL-C value was considered missing, as the Friedewald formula became invalid in such cases. Dyslipidemia was defined as having at least one lipid parameter in the abnormal range, i.e., TC ≥ 240 mg/dL, LDL-C ≥ 160 mg/dL, HDL-C < 40 mg/dL in males or <50 mg/dL in females, or TG ≥ 200 mg/dL, according to the Guideline on the Management of Blood Cholesterol developed by American Heart Association and other organizations in 2018 [23].

### 2.3. Statistical Analysis

The demographic characteristics of participants were described with counts and weighted percentages (for categorical variables) or weighted means with standard deviations (for continuous variables). The primary results were the proportions of different combinations of the four lipid abnormalities among individuals with dyslipidemia. Statistical analysis was performed utilizing the survey package (version 4.2-1) in R environment (version 4.2.1). The “survey” package is widely used in the analysis of weighted data. NHANES has also recommended using the “survey” package for data analysis and provided sample codes to account for the complex sampling design, oversampling, and non-response. Fasting sample weights were used in the study as LDL-C and TG levels were measured only in individuals who completed fasting. A 20-year weight was constructed with 4-year weights for the 1999–2002 data and 2-year weights for the 2003–2018 data. The differences in prevalence of lipid abnormalities across demographic characteristics (i.e., age, sex, race) and time period were assessed by χ^2^ test. Trend tests for age group and time period were conducted by a weighted logistic regression. Area-proportional Euler diagrams were generated to show the overlapping pattern using the “Eulerr” package (version 7.0.0).

## 3. Results

**Figure 1:**
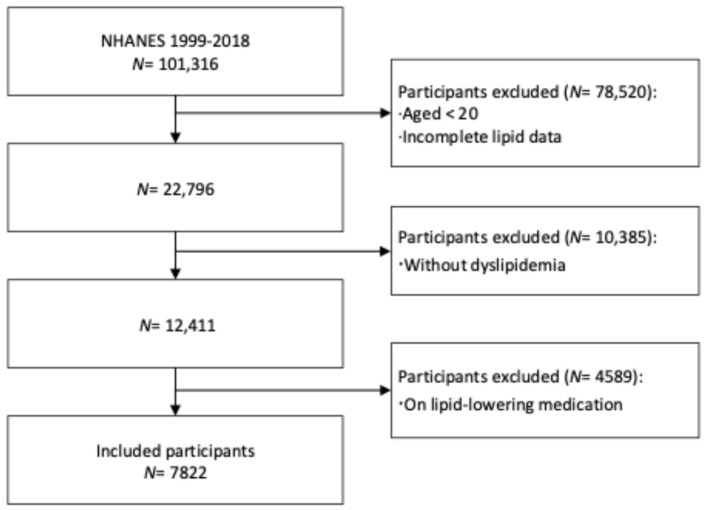
Flow diagram of inclusion and exclusion of participants.

**Figure 2:**
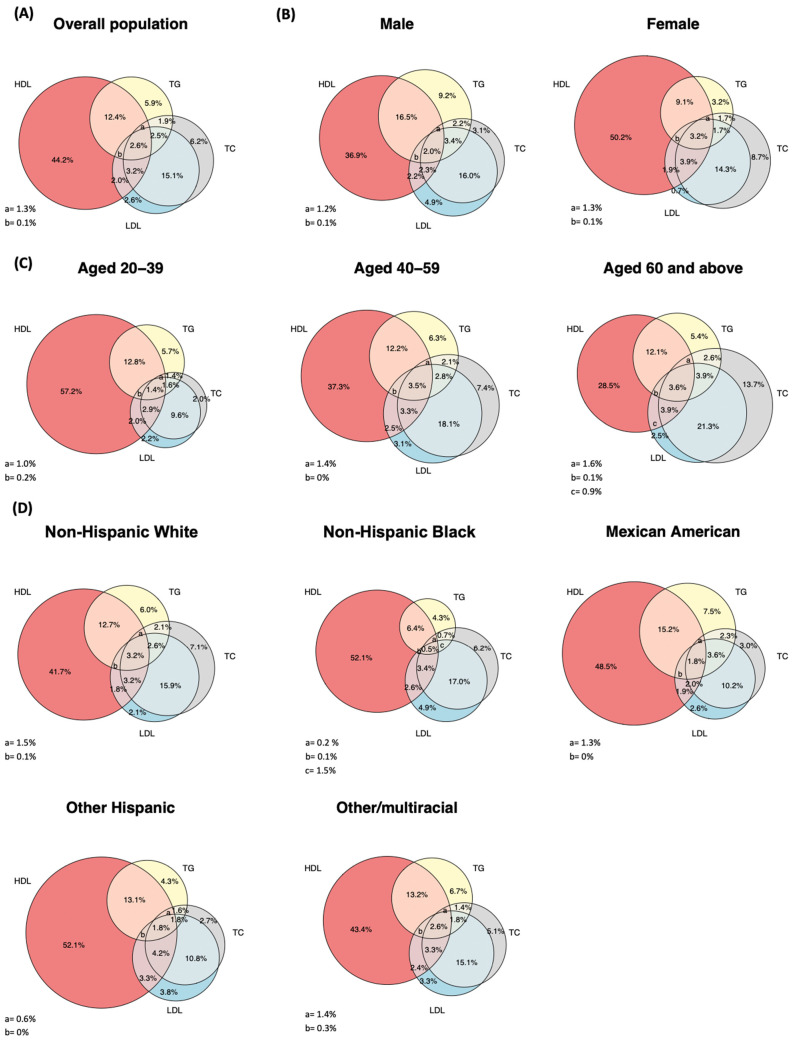
Weighted percentage of people with different combinations of high LDL-C, high TC, high TG, and low HDL-C among participants with dyslipidemia but not on medications in the overall population (**A**); stratified by sex (**B**); stratified by age group (**C**); and stratified by race (**D**). The percentages may not add up to 100% because of rounding.

The flow of the inclusion and exclusion of participants is shown in Figure 1. Among the participants aged 20 years or above in the 10 NHANES cycles from 1999–2000 to 2017–2018, 22,796 had complete data on all four lipid parameters, and 52.1% of them (*N* = 12,411) had dyslipidemia. After excluding those on lipid-lowering drug treatments, 7822 participants (66.1% of all people with dyslipidemia) were eventually included in this study. Table 1 shows the basic characteristics of the study participants. Of the participants, those with high TC, high LDL-C, high TG, and low HDL-C accounted for 32.8% (95% confidence interval [CI]: 31.3%–34.2%), 28.1% (95% CI: 26.6%–29.6%), 26.7% (95% CI: 25.4%–28.0%), and 65.9% (95% CI: 64.0%–67.7%), respectively (Figure 2A). Notably, the most prevalent lipid abnormality across all subgroups was low HDL-C (Figure 2B–D).

Individuals categorized as “high LDL-C or high TC” (irrespective of HDL-C and TC levels), “normal LDL-C, normal TC, but high TG” (irrespective of HDL-C level), and “normal LDL, normal TC, normal TG, but low HDL-C” (i.e., isolated HDL-C) accounted for 37.5% (95% CI: 35.9%–39.1%), 18.3% (95% CI: 17.2%–19.4%), and 44.2% (95% CI: 42.5%–46.0%) of the total, respectively (Table 2). The total proportion of those who had “normal LDC-C, normal TC, but high TG and/or low HDL-C” out of all people with dyslipidemia was similar (61.1%–63.2%) across different periods (Table 2). The proportion of “high LDL-C or high TC” increased significantly with age and accounted for 54.0% (95% CI: 50.9%–57.1%) of all dyslipidemia in people aged ≥60 years (Table 2). Supplementary analysis revealed that those with TG levels exceeding 400 mg/dL, 500 mg/dL, and 1000 mg/dL, which are believed to be associated with a heightened risk of acute pancreatitis, constituted 2.0% (95% CI: 1.8%–2.2%), 1.1% (95% CI: 0.9%–1.3%), and 0.2% (95% CI: 0.1%–0.3%) of the adult population, respectively.

## 4. Discussion

Statin therapies for lowering LDL-C levels have proven effective in reducing the risks of CVDs and mortality [32,33], and are currently recommended as a first-line treatment for a broad spectrum of individuals for both primary and secondary prevention of CVD [23]. Since LDL-C level is the main determinant of TC level, the two lipid components are typically highly correlated with each other. For example, in this study, among those with high LDL-C, 83% also had high TC. Among the other 17% who had elevated LDL-C levels but normal TC levels, 7.6% had low HDL-C levels. Therefore, individuals with high LDL-C and those with high TC are expected to derive similar cardiovascular benefits from lipid-lowering treatments, irrespective of their HDL-C and TG levels. For this reason, LDL-C and TC are commonly evaluated together in clinical practice. Our findings indicated that 37.5% of all individuals with dyslipidemia belong to this subset and could potentially benefit from taking LDL-lowering drugs in terms of CVD prevention. Evidence from randomized trials showed that such medications can prevent 25 (95% CI, 19–31) major cardiovascular events among every 1000 treated individuals in the primary prevention setting and 48 (95% CI, 39–57) events in the secondary prevention setting [33]. Assuming (1) a total adult population aged ≥ 20 years in the United States of 217,270,203, according to the sample weight adopted by NHANES [34], (2) a dyslipidemia prevalence of 52.1%, as estimated in this study, and (3) all adults with high LDL-C or TC (37.5% of all dyslipidemia) receiving lipid-lowering medications had high adherence rates, the number of major cardiovascular events that could be prevented by drug treatments of dyslipidemia in the United States over a 5-year period would be between 2,462,052 and 3,735,527. While recognizing the benefit of lipid-lowering statin treatment, it should be noted that the treatment can cause adverse effects in skeletal muscle, liver function, and the nervous system [35,36]. Statin-associated muscle symptoms are the most common, affecting up to 25% of the treated patients [37]. Furthermore, regular treatment with statins represents a huge financial burden to the healthcare system, costing $10 billion in total and $3.1 billion out of pocket every year among the patients who reported taking statin therapy in the United States [38]. The potential adverse effects, cost, and perceived benefit of treatment, individually or jointly, may lead to a low adherence rate or even discontinuation of treatment. A previous study showed that only 36.8% of the patients without coronary heart disease or diabetes mellitus strongly adhered to the treatment, constituting a big challenge to the primary prevention of CVDs [39].

Another finding of this study was that low HDL-C contributed most (65.9%) to the prevalence of dyslipidemia, with isolated low HDL-C accounting for 44.2% of all dyslipidemia cases. Observational studies have consistently demonstrated that low HDL-C was associated with a higher risk of CVDs after adjusting for traditional non-lipid risk factors [40,41]. However, randomized trials found that drug treatment aiming to increase HDL-C levels did not reduce CVDs [21,22]. One possible explanation for this discrepancy is that HDL-C may not fully represent HDL functionality. It is widely believed that HDL-C can lower the risk of atherosclerosis by promoting cholesterol efflux, which reduces cholesterol accumulation in the macrophages and thereby can lower the risk of atherosclerosis by promoting cholesterol efflux, facilitating lipid oxidation, enhancing endothelial function, anti-inflammation, and anti-apoptosis [42]. While HDL-C is indeed correlated with HDL function and can be used to estimate the cholesterol levels within HDL particles, recent studies suggest that the number of HDL particles (HDL-P) per se is a better indicator of HDL functionality. HDL-P demonstrates strong associations with HDL-C function, including cholesterol transport and antioxidation [43,44]. Moreover, the association of HDL-C with CVD becomes statistically insignificant after adjusting for HDL-P, whereas the association of HDL-P with CVD persists even after adjusting for HDL-C [44]. As the HDL-enhancing drugs evaluated in previous randomized trials primarily aimed to boost HDL-C levels rather than HDL-P levels, it was not surprising that they were not associated with the reduction in CVD. Given this evidence, the current AHA/ACC guidelines do not recommend initiating statin therapies based on isolated low HDL-C levels, but acknowledge that low HDL-C level is a risk-enhancing factor that should be considered during the clinician–patient discussion on whether to initiate statin therapy [23]. The US Preventive Services Task Force recommends initiating statin therapies in adults aged 40 to 75 years without a history of CVD and with one or more cardiovascular risk factors, if their 10-year CVD risk exceeds 7.5% [25].

The effects of TG on cardiovascular risk are also ambiguous. Despite numerous epidemiological studies identifying high TG as a risk factor for CVD [45,46,47,48], randomized trials evaluating TG-lowering drug treatments for CVD prevention yielded conflicting results, with only one trial, i.e., the REDUCE-IT study, reporting a statistically significant reduction in CVD [20]. Although individuals with very high TG may benefit from TG-lowering treatment to prevent acute pancreatitis [49,50,51], this study revealed that only a small proportion of individuals with dyslipidemia had a higher TG level (≥400 mg/dL, 3.3%; ≥500 mg/dL, 1.8%; 1000 mg/dL, 0.4%), which was associated with an increased risk of acute pancreatitis. This suggests that the majority of individuals with high TG levels are less likely to derive a significant benefit from such treatments. The 2018 ACC/AHA guidelines recommend initiation of statin therapy only when elevated TG (≥170 mg/dL) persists after tackling lifestyle factors, secondary factors, and potential TG-raising medications [23]. Overall, individuals with isolated low HDL-C or isolated high TG (and normal LDL-C level) are less likely to benefit from lipid-lowering drug treatment to further reduce their LDL-C levels. The 2019 ESC/EAS guidelines (page 126) suggest that “Class I recommendation, Level A evidence” for such treatment is only available for those with a 10-year CVD risk > 10%, which represents a small fraction of all individuals [25,26]. This highlights the need for further research and the development of new drugs that are targeted at isolated high TG and/or low HDL-C and can effectively reduce CVD risk.

In addition to the four lipid measures examined in this study, non-high-density lipoprotein (non-HDL-C), which is calculated by subtracting HDL-C levels from TC levels, is another risk factor for CVD and gaining increasing attention. Previous studies showed that it was associated with increased CVD risk even after adjusting for LDL-C level in the model [52,53]. In the present study, the subjects had abnormalities in at least one of the four routinely assessed lipid components, and therefore none of them had isolated elevated non-HDL-C. Our exploratory analysis showed that, among NHANES participants with measurements of all the four lipid components (normal or abnormal) and not on lipid-lowering medications, 41.5% demonstrated isolated elevated non-HDL-C levels, 18.5% isolated low HDL-C levels, and 10.6% both. Again, this calls for more research on individuals with isolated low HDL-C as their CVD risk may be increased due to their non-HDL-C levels rather than the low HDL-C alone and there are currently no particular recommendations regarding treatment for this subgroup.

The strengths of this study include its utilization of NHANES data—which makes the results generalizable to the general adult population of the United States—and the comprehensive analyses on the overlapping patterns of dyslipidemia components across different subgroups and periods. However, it is important to interpret the findings of this study with some caution. Firstly, only the participants who were not on lipid-lowering medications were included, who may not represent the overall population with dyslipidemia. A comparison of the NHANES participants with dyslipidemia on medications versus those not on medications (Table A1) showed that the former were more likely to be male, older, and non-Hispanic. However, this issue is unavoidable because the lipid levels of participants on medications had been altered and would introduce bias if they were included in the analysis. Secondly, LDL-C in this study was not an independently measured parameter but estimated by using the Friedewald formula. The “TG/5” in the formula reflects the cholesterol in TG-rich ApoB lipoproteins, very-low-density lipoprotein, and intermediate-density lipoproteins. The error in estimation may determine whether the LDL-C values end up just below or just above the limit of the normal range and thus cause the misclassification of some individuals. Furthermore, the participants with a TG level > 400 mg/dL were excluded from the estimation. However, as shown by our supplementary analysis, these participants represented a very small fraction of all people with dyslipidemia and were unlikely to have a large impact on the main results of this study, e.g., the overlapping pattern of individual lipid abnormalities. Thirdly, the use of lipid-lowering medication was self-reported, which in theory was subject to recall bias. However, a previous report from the Atherosclerosis Risk in Communities Study demonstrated a high level of accuracy of self-reported lipid-lowering medication use, with 89% to 99% of the people who self-reported not taking medications truly not on medications, as verified by the information on inventoried lipid medication containers [54]. Thus, the bias arising from self-report, if any, was likely small.

## 5. Conclusions

Among the individuals with dyslipidemia, only 37.5% would gain proven benefits from currently available medications in terms of LDL-C or TC reduction. Individuals with isolated low HDL-C or isolated high TG, who are less likely to benefit from existing drug treatments in terms of CVD prevention, account for around 60% of the dyslipidemia population. Although some of them could benefit from drug treatment to further reduce their LDL-C levels, they represent a small fraction of individuals. These findings have implications for the precise management of dyslipidemia, resource allocation for primary prevention of CVD, and drug discovery and development in the future.

## Figures and Tables

**Table 1:** Basic characteristics of the participants included in the study (*n* = 7822).

Characteristics	No. (Weighted %) or Mean (Standard Deviation)
Sex, *n* (%)	
Male	3405 (45.0)
Female	4417 (55.0)
Age, *n* (%) *	
20–39 years	3062 (42.2)
40–59 years	2762 (40.3)
≥60 years	1998 (17.4)
Race, *n* (%) *	
Non-Hispanic White	3409 (66.9)
Non-Hispanic Black	1307 (9.9)
Mexican American	1707 (10.0)
Other Hispanic	744 (6.8)
Other/multiracial	655 (6.5)
Lipid, mean (standard deviation), mg/dL	
TC	212.01 (47.9)
LDL-C	133.28 (39.8)
HDL-C	46.95 (15.7)
TG	158.91 (75.5)

Abbreviations: TC, total cholesterol; LDL-C, low-density lipoprotein cholesterol; HDL-C, high-density lipoprotein cholesterol; TG, triglycerides. * The percentages do not add up to 100% because of rounding.

**Table 2:** Weighted percentage and 95% CI of different combinations of high LDL-C, high TC, high TG, and low HDL-C among the participants with dyslipidemia but not on medications.

	High LDL-C or High TC	Normal LDL-C, Normal TC, but High TG	Normal LDL, Normal TC, Normal TG, but Low HDL-C	Normal LDC-C, Normal TC, but High TG and/or Low HDL-C
Overall	37.5 (35.9–39.1)	18.3 (17.2–19.4)	44.2 (42.5–46.0)	62.5 (60.9–64.1)
Sex				
Male	37.4 (35.1–39.7)	25.7 (23.9–27.6)	36.9 (34.5–39.4)	62.6 (60.3–64.9)
Female	37.6 (35.5–39.7)	12.2 (11.1–13.5)	50.2 (48.2–52.2)	62.4 (60.3–64.5)
*p*-Value	0.873	<0.001	<0.001	0.873
Age				
20–39	24.3 (22.3–26.4)	18.4 (16.7–20.3)	57.2 (54.8–59.7)	75.7 (73.6–77.7)
40–59	44.1 (41.7–46.6)	18.5 (16.7–20.5)	37.3 (35.0–39.8)	55.9 (53.4–58.3)
≥60	54.0 (50.9–57.1)	17.4 (15.4–19.7)	28.5 (25.9–31.3)	46.0 (42.9–49.1)
* p * for trend	<0.001	0.494	<0.001	<0.001
Race				
Non-Hispanic White	39.6 (37.5–41.8)	18.7 (17.3–20.2)	41.7 (39.4–44.0)	60.4 (58.2–62.5)
Non-Hispanic Black	37.2 (34.4–40.0)	10.7 (8.9–12.8)	52.1 (49.2–55.0)	62.8 (60.0–65.6)
Mexican American	28.8 (25.8–32.0)	22.7 (20.1–25.4)	48.5 (45.3–51.8)	71.2 (68.0–74.2)
Other Hispanic	30.5 (26.6–34.7)	17.4 (14.6–20.6)	52.1 (47.8–56.4)	69.5 (65.3–73.4)
Other/multiracial	36.6 (31.3–42.4)	19.9 (15.7–25.0)	43.4 (37.4–49.6)	63.4 (57.6–68.7)
*p*-Value	<0.001	<0.001	<0.001	<0.001
Time period				
1999–2004	36.8 (34.0–39.6)	20.5 (18.5–22.6)	42.7 (39.6–45.9)	63.2 (60.4–66.0)
2005–2010	38.9 (36.1–41.7)	18.9 (17.2–20.8)	42.2 (39.1–45.4)	61.1 (58.3–63.9)
2011–2018	37.1 (34.2–40.1)	15.9 (14.1–17.8)	47.1 (44.1–50.0)	62.9 (59.9–65.8)
* p * for trend	0.887	0.001	0.047	0.887

Abbreviations: TC, total cholesterol; LDL-C, low-density lipoprotein cholesterol; HDL-C, high-density lipoprotein cholesterol; TG, triglycerides.

## Data Availability

The datasets are publicly available at the National Center for Health Statistics of the Center for Disease Control and Prevention (https://www.cdc.gov/nchs/nhanes/, accessed on 17 April 2024).

## Appendix A

**Table A1:** Basic characteristics of NHANES participants with dyslipidemia by medication use status.

Characteristics	No. (%)		*p*-Value
	On Medication (*N* = 4589)	Not on Medication (*N* = 7822)	
Gender, *n* (%)			<0.001
Male	2400 (51.9)	3405 (45.0)	
Female	2189 (48.1)	4417 (55.0)	
Age, *n* (%)			<0.001
20–39 years	96 (2.9)	3062 (42.2)	
40–59 years	1165 (35.5)	2762 (40.3)	
≥60 years	3328 (61.6)	1998 (17.4)	
Race, *n* (%)			<0.001
Non-Hispanic White	2368 (76.3)	3409 (66.9)	
Non-Hispanic Black	877 (9.2)	1307 (9.9)	
Mexican American	571 (4.3)	1707 (10.0)	
Other Hispanic	375 (3.4)	744 (6.8)	
Other/multiracial	398 (6.8)	655 (6.5)	
